# Joint and separate exposure to alcohol and ∆^9^-tetrahydrocannabinol produced distinct effects on glucose and insulin homeostasis in male rats

**DOI:** 10.1038/s41598-019-48466-w

**Published:** 2019-08-19

**Authors:** Nnamdi G. Nelson, Michael J. Weingarten, Wen Xuan Law, Daniel T. Sangiamo, Nu-Chu Liang

**Affiliations:** 1 Neuroscience Program, University of Illinois at Urbana-Champaign, Champaign, USA; 2 Department of Psychology, University of Illinois at Urbana-Champaign, Champaign, USA

**Keywords:** Obesity, Experimental models of disease

## Abstract

Cannabis and alcohol co-use is common, and the trend may increase further given the current popularity of cannabis legalization. However, the metabolic consequences of such co-use are unclear. Here, we investigated how co-administration of alcohol and ∆^9^-tetrahydrocannabinol (THC), the main psychoactive constituent of cannabis, affects body weight and visceral adiposity, and glucose and insulin homeostasis in rats. For 16 consecutive days during adolescence, male rats drank saccharin or alcohol after receiving subcutaneous oil or THC injections in Experiment 1 and voluntarily consumed alcohol, THC edible, or both drugs in Experiment 2. Experiment 1 showed that following abstinence, drug co-exposure reduced visceral fat and the amount of insulin required to clear glucose during an oral glucose tolerance test (OGTT). In Experiment 2, rats received a high-fat diet (HFD) after 3-week abstinence. Although adolescent drug use did not interact with the HFD to worsen hyperglycemia and hyperinsulinemia during an OGTT, HFD-fed rats that co-used alcohol and THC had the lowest insulin levels 75 min after an insulin injection, suggesting an altered rate of insulin secretion and degradation. These results suggest that THC and alcohol co-exposure can distinctly alter the physiology of glucose and insulin homeostasis in a rodent model.

## Introduction

The wide prevalence and burdensome nature of obesity and metabolic syndrome echoes the need for more studies to shed light on the predisposing and protective factors at play. Previous studies have established that early-life nutritional deficits or surfeits can influence neurological and metabolic health throughout the lifespan^1^, as does active or passive exposure to exogenous drugs like alcohol and cannabinoids^2,3^. Prolonged consumption of diets rich in calories, salt, saturated fatty acids, and refined sugars but low in fiber (“Western diet”) coupled with a sedentary lifestyle are notable contributors to the overweight/obese phenotype^4^. The contribution of exogenous drugs, especially alcohol and marijuana co-use, to the high rate of metabolic disorders has been scarcely investigated.

Use and co-use of alcohol and marijuana often begin during adolescence^5^. This drug use behavior is worrisome considering the wealth of empirical evidence suggesting that the adolescent brain is susceptible to the influence of exogenous compounds^6,7^. Notably, alcohol or cannabis use during adolescence can alter the ontogeny of neural systems that regulate cognitive and reward-related behaviors^8,9^ to provoke aberrant brain functioning later in life^10,11^. Similar neural alterations can translate into an increased motivation to consume palatable, energy-dense diets during young adulthood^12,13^. Despite the popularity of alcohol and cannabis co-use^14^, there is paucity of studies on how such co-use affects energy balance and metabolic outcomes during abstinence.

Epidemiological data have implicated chronic alcohol use with abdominal adiposity and some facets of metabolic syndrome, including leptin and insulin resistance, and type-2 diabetes^15^. Other researchers conclude that moderate alcohol consumption may either be beneficial to cardio-metabolic health or have null effects^16^. Meanwhile, few human studies reported the metabolic effects of marijuana use^17,18^, and there has been no preclinical investigation on this topic. Human laboratory studies have documented that marijuana smoking can acutely increase plasma insulin concentration and alter glucose tolerance^19,20^ or have no effect on glucose homeostasis^21^ in adult subjects. A cross-sectional, case-control study reported that chronic marijuana smoking can reduce circulating HDL-cholesterol and increase adipose tissue hypertrophy and insulin resistance without significant detriment to pancreatic β-cell function^22^. These reports suggest that repeated cannabis use may alter insulin control of glucose homeostasis. Importantly, given that alcohol and cannabis co-administration is common and may be on the rise due to the current social atmosphere of cannabis legalization, how alcohol and ∆^9^-tetrahydrocannabinol (THC), the main psychoactive constituent of cannabis, interact to affect energy balance and glucose homeostasis deserves investigation.

**Figure 1:**
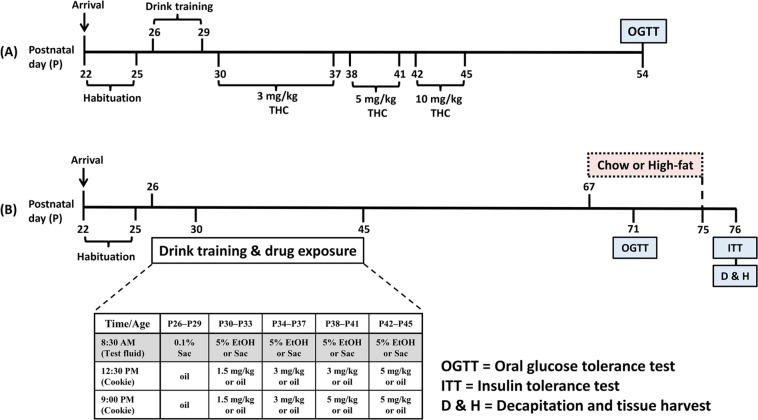
(**A**) Timeline of chronic subcutaneous THC injection and voluntary alcohol drinking in Experiment 1 (EtOH, n = 8; CTL, THC, and COM, n = 10/group). The 3 mg/kg/day THC lasted for eight days, while the 5 and 10 mg/kg/day THC each lasted for four days. An oral glucose tolerance test (OGTT) was performed on P54 after an overnight fast. (**B**) Timeline of chronic oral THC consumption and voluntary alcohol drinking in Experiment 2. The table indicates the schedules and duration for drinking and drug exposure. The shaded portion of the table indicates the last 3 h of the dark cycle when access to the test fluid occurred. From P67, some rats continued consuming regular chow diet while the others received 45% high-fat diet. Glucose and insulin tolerance tests were performed on P71 and P76, respectively [Chow (CTL, n = 14; EtOH, n = 12; THC, n = 10; COM, n = 11) and HF (CTL and COM, n = 11/group; EtOH and THC, n = 12/group)].

Previously, we demonstrated that male rats that chronically consumed moderate alcohol doses showed early signs of insulin insensitivity^23^. The lack of systematic investigation of the lasting behavioral and metabolic effects of alcohol and THC co-administration prompted us to undertake this study using a rodent model of adolescent exposure of the two drugs^24^. Given the negative metabolic outcomes of short-term Western diet consumption in young subjects^25,26^, we sought to investigate whether high-fat diet (HFD) exposure would exacerbate the impact of prior alcohol and THC use. We hypothesized that, compared to separate administration of alcohol and THC, their co-administration during adolescence would lead to lasting alterations in feeding behavior that will prime the subject for heightened reactivity to metabolic challenges in adulthood. Hence, we predicted that rats previously co-exposed to alcohol and THC would demonstrate hyperphagia and increased adiposity when presented with an HFD compared with the drug naïve rats. We also expected that combined drug co-use and HFD overconsumption would greatly impair glucose tolerance and insulin sensitivity during adulthood. Accordingly, Experiment 1 investigated how repeated alcohol drinking, subcutaneous THC injection, or combinational exposure of the two drugs during adolescence affect metabolic measures e.g., body fat, blood glucose and insulin responses to an oral load of glucose challenge. Consumption of edibles prepared with marijuana extract is popular among medical and recreational marijuana users^27^. Thus, Experiment 2 investigated how adolescent voluntary consumption of alcohol and THC edibles, alone or in combination, affect feeding and metabolic responses to HFD during early adulthood (See Fig. 1 for experimental timeline).

## Results

### Experiment 1: Results

#### Combined subcutaneous THC injection and alcohol consumption reduced visceral fat composition

**Figure 2:**
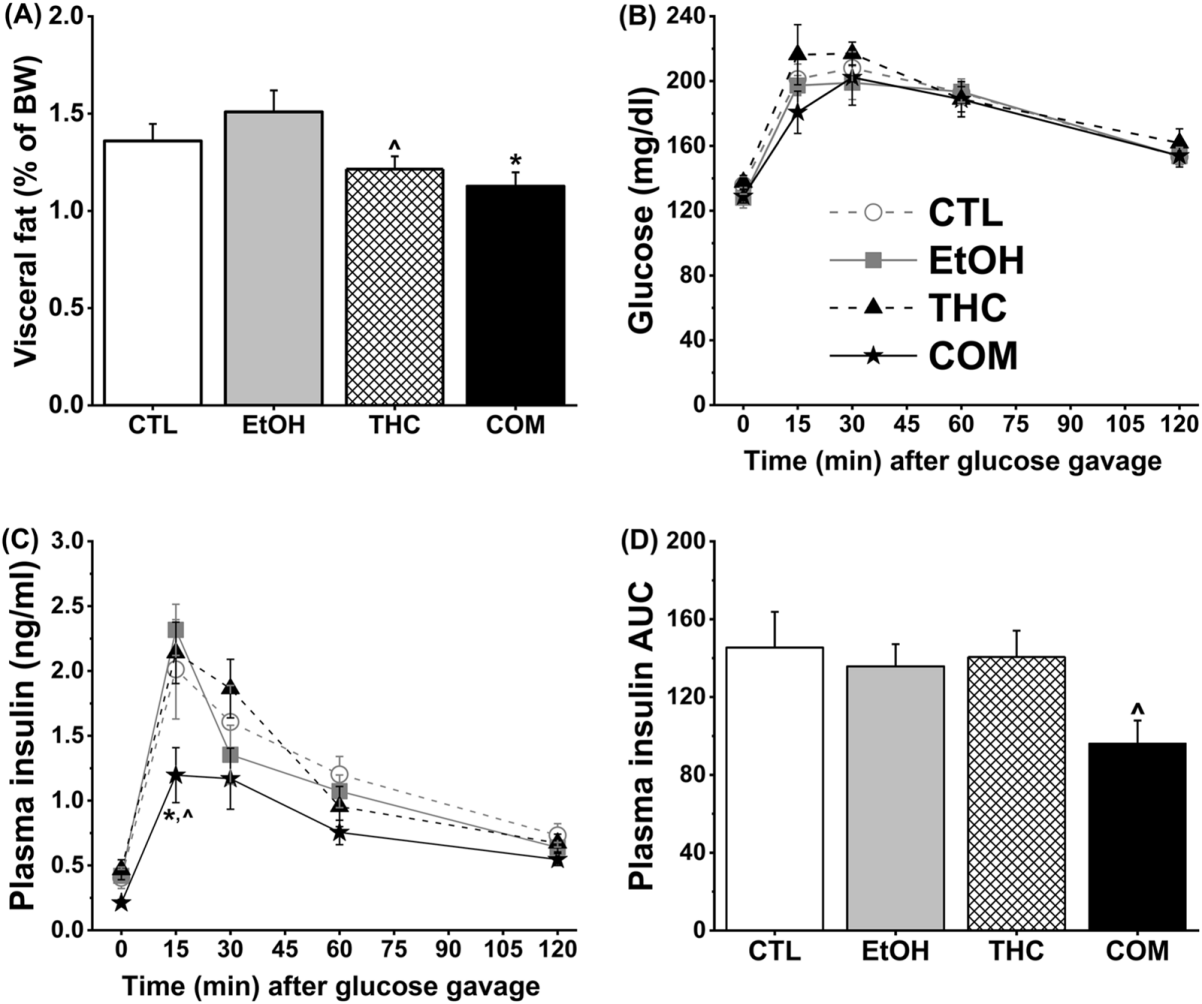
Chronic subcutaneous THC injections and voluntary alcohol consumption differently affected visceral fat composition and reduced insulin levels needed to clear glucose in Experiment 1 (CTL, n = 9; EtOH and THC, n = 7; COM, n = 8). (**A**) THC alone or in combination with alcohol reduced visceral fat composition. THC vs. EtOH: ^*p* < 0.1; COM vs. EtOH: *p < 0.009. The OGTT was conducted after nine days of abstinence. (**B**) Alcohol and THC alone or in combination had no effect on glucose clearance; and (**C**) combined chronic alcohol and THC exposure was associated with reduced plasma insulin levels at 15 min following the oral glucose load. COM vs. EtOH: *p < 0.009 and COM vs. THC: ^*p* < 0.07. (**D**) The COM group had a reduced AUC of plasma insulin curve compared with the CTLs (*t*-test: ^*p* < 0.05).

Alcohol, food intake, and weight gain during drug treatment and abstinence are presented in our previous published manuscript^24^. In brief, THC dose-dependently increased food intake during the initial 3 h after subcutaneous injections but did not alter alcohol consumption and blood ethanol concentration (BEC) during the same period. Voluntary alcohol consumption acutely reduced food intake when it was consumed alone (EtOH group) or when THC was on-board in the COM group. Further, THC or alcohol had no effect on daily caloric intake and weight gain during treatment. However, the COM group gained the least amount of weight during the week of abstinence compared with the EtOH and THC groups^24^. On the eighth day of abstinence (P53), the respective body weights of the CTL (241.7 ± 3.5 g), EtOH (248.4 ± 3.5 g), THC (238.2 ± 6.1 g), and COM (234.0 ± 4.4 g) groups were statistically indistinguishable. Yet, the COM group had lower percent visceral fat on P54 compared with the EtOH group [*F*_(3,34)_ = 4.41, *p* < 0.02; post hoc, *p* < 0.009; Fig. 2A].

#### Combined subcutaneous THC injection and alcohol consumption reduced plasma insulin concentration following an oral load of glucose

The 16 days of subcutaneous THC and moderate alcohol treatment did not alter fasting blood glucose levels (Fig. 2B). All of the groups of rats comparably cleared blood glucose across time during the OGTT [time effect, *F*_(4,136)_ = 94.95, *p* < 0.0001; Fig. 2B]. The fasting plasma insulin concentration of the COM group was mildly lower than that of the THC group [group effect, *F*_(3,27)_ = 3.00, *p* < 0.05; post hoc, *p* < 0.06; Fig. 2C]. Rats in each group had a slightly different profile of plasma insulin concentration during the 2 h following the intragastric 2.0 g/kg glucose load [group, time, and group × time effects: *F*_(3,27)_ = 2.58, *p* < 0.08, *F*_(4,108)_ = 73.90, *p* < 0.0001, and *F*_(12,108)_ = 2.01, *p* < 0.03, respectively]. At the 15-min time point, the plasma insulin level of the COM group was lower than that of both the EtOH (*p* < 0.009) and THC (*p* < 0.07) groups. Finally, the COM group demonstrated a trend for an overall reduced insulin response compared with the CTLs [insulin AUC: group effect, *F*_(3,27)_ = 2.45, *p* < 0.09; post hoc, *p* < 0.09; Fig. 2D]. In support of our *a priori* hypothesis that drug combination will elicit unique effects, independent samples *t*-test revealed that the area under the insulin curve of the COM group was significantly lower than that of the CTLs [*t*_(15)_ = 2.19, *p* < 0.05].

### Experiment 2: Results

#### Male rats readily consumed THC-laced cookies

Most rats consumed the THC-laced cookie within minutes. Two and four rats from the THC and COM groups, respectively, had bits of leftover cookies by the time a second cookie was delivered. The subsequent data of these rats were excluded from analyses.

#### Consumption of THC edible reduced intakes of saccharin but not alcohol

**Figure 3:**
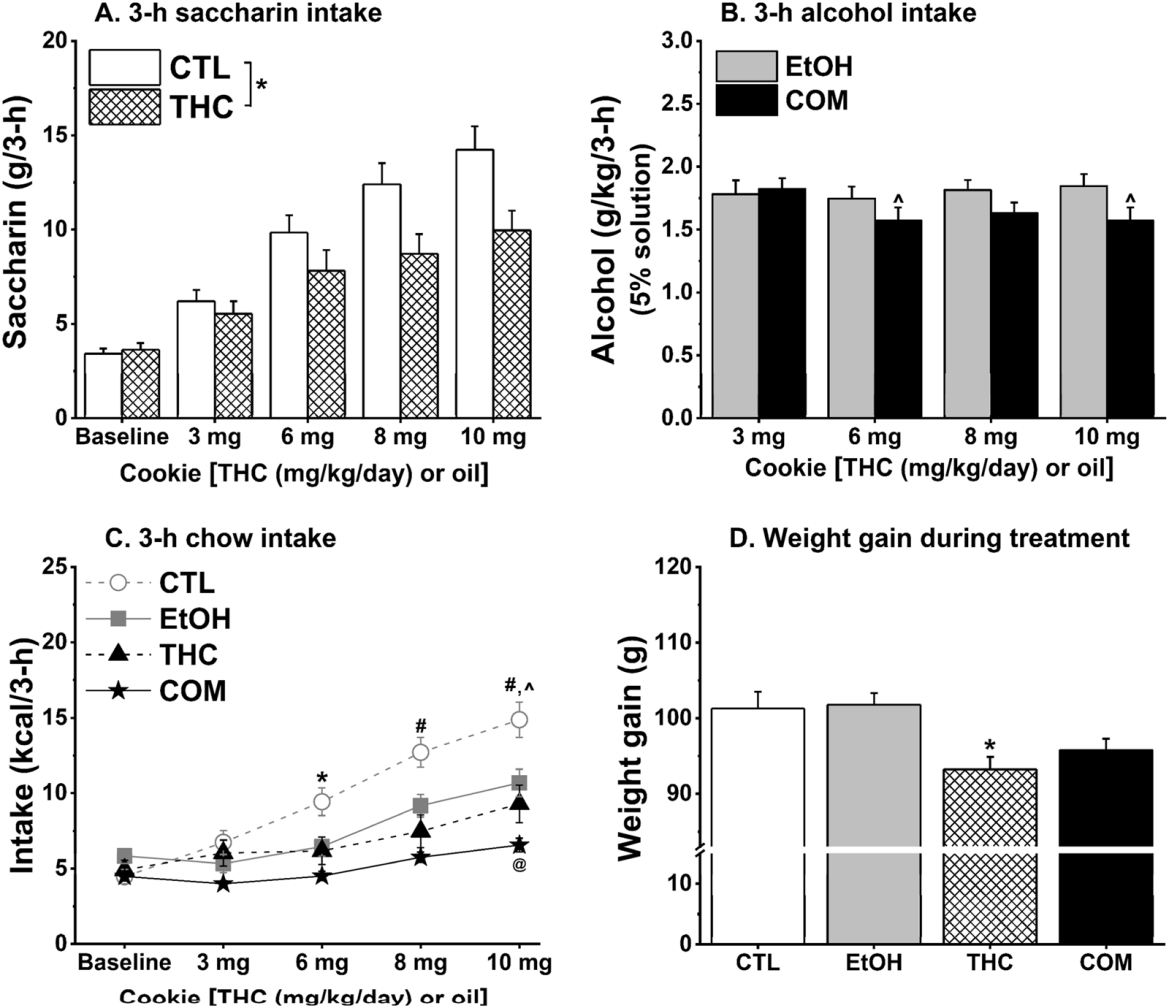
Chronic oral THC consumption reduced saccharin intake and chow intake during the last 3 h of the dark cycle and weight gain, whereas moderate alcohol alone or when combined with oral THC reduced 3-h chow intake (CTL, n = 25; EtOH, n = 24; THC and COM, n = 22/group). (**A**) THC rats consumed less saccharin compared with the CTL rats. *p < 0.04. (**B**) EtOH and COM rats consumed similar doses of alcohol during each THC dose. COM rats’ alcohol intake during 6 and 10 mg/kg/day THC consumption was lower than their intake during 3 mg/kg/day THC consumption: ^*p* < 0.03. (**C**) Drinking 5% alcohol reduced 3-h chow intake and augmented the somewhat hypophagic effect of oral THC. CTL vs. COM: *p < 0.005; CTL vs. THC and COM: ^#^*p* < 0.004; CTL vs. EtOH: ^*p* < 0.06; EtOH vs. COM: ^@^*p* < 0.03. (**D**) Oral THC suppressed weight gain in the THC group compared with both the CTL and EtOH groups: *p < 0.02.

The CTL and THC rats consumed increasing amounts of saccharin across treatment days [*F*_(3,135)_ = 78.37, *p* < 0.0001]. But the THC rats consumed less saccharin compared with the CTLs [*F*_(1,45)_ = 4.93, *p* <= 0.04; Fig. 3A]. The EtOH and COM groups consumed similar doses of alcohol across treatment days (Fig. 3B). Despite no group effect on alcohol intake, COM rats appeared to reduce alcohol intake during consumption of higher doses of THC (6 and 10 mg/kg/day). On the days BECs were measured, EtOH (1.36 ± 0.07 g/kg) and COM (1.20 ± 0.08 g/kg) rats consumed similar doses of alcohol within 1 h following 8 mg/kg/day THC consumption. Both groups of rats attained comparable BECs of 25.50 ± 4.08 mg/dl and 25.95 ± 5.67 mg/dl, respectively. After 3 h of alcohol access following 10 mg/kg/day THC consumption, EtOH (1.65 ± 0.10 g/kg) and COM (1.45 ± 0.13 g/kg) rats consumed comparable alcohol doses and attained BECs of 13.57 ± 3.63 mg/dl and 18.35 ± 5.30 mg/dl, respectively. The BECs attained after both 1- and 3-h alcohol drinking significantly correlated with the ingested dose in both the EtOH [*r* = 0.74, *p* < 0.0002 and *r* = 0.64, *p* < 0.008, respectively] and COM [*r* = 0.65, *p* < 0.004 and *r* = 0.91, *p* < 0.0001, respectively] groups.

#### Adolescent alcohol and THC co-use facilitated weight gain in response to HFD during early adulthood

Consumption of THC-laced cookies on the previous day blunted chow intake during the last 3 h of the dark cycle on the next day in the THC and COM groups relative to the CTLs [*F*_(3,89)_ = 8.46, *p* < 0.0001; post hoc, both *p* < 0.007; Fig. 3C]. The EtOH group consumed less 3-h chow compared with the CTL group (*p* < 0.04). The lower 3-h chow intake by THC and COM rats compared with the CTLs was retained after intake was normalized to body weight. There were no group differences in daily caloric intake during the 16-day treatment period, but the THC rats gained less weight than did the EtOH and CTL rats [*F*_(3,89)_ = 5.39, *p* < 0.002; post hoc, both *p* < 0.02; Fig. 3D].

**Figure 4:**
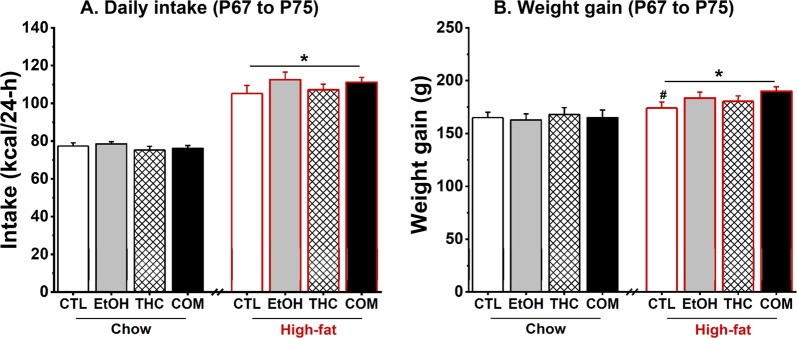
Chronic oral THC and voluntary alcohol consumption, alone or when combined, had no effect on daily caloric intake and weight gain during abstinence (from P67–P75) [Chow (CTL, n = 14; EtOH, n = 12; THC, n = 10; COM, n = 11) and HFD (CTL and COM, n = 11/group; EtOH and THC, n = 12/group)]. (**A**) Rats maintained on 45% HFD consumed more average daily calories compared with intake by those maintained on regular chow diet: *p < 0.0001. (**B**) HFD-fed rats gained more weight compared with the chow-fed rats: *p < 0.0001 by ANOVA. Under the HFD condition, the COM rats gained more weight compared with the CTLs: ^#^*p* < 0.04 by *t*-test.

Compared with chow-fed controls, rats that consumed 45% HFD for four days (P67–P70) demonstrated hyperphagia [*F*_(1,38)_ = 412.58, *p* < 0.0001] and modest weight gain [*F*_(1,38)_ = 5.11, *p* < 0.03]. There were no drug effects on daily chow or HFD intake during the 9-day test period. As expected, the HFD-fed rats consumed approximately 30% more daily calories [*F*_(1,38)_ = 252.48, *p* < 0.0001; Fig. 4A] and gained more weight [*F*_(1,38)_ = 25.23, *p* < 0.0001; Fig. 4B] compared with the chow-fed rats. Under the HFD condition, the COM group gained more weight than the CTLs did [*t*_(20)_ = −2.21, *p* < 0.04].

#### Adolescent THC and alcohol co-use did not affect glucose and insulin tolerance following short-term HFD challenge during early adulthood

**Figure 5:**
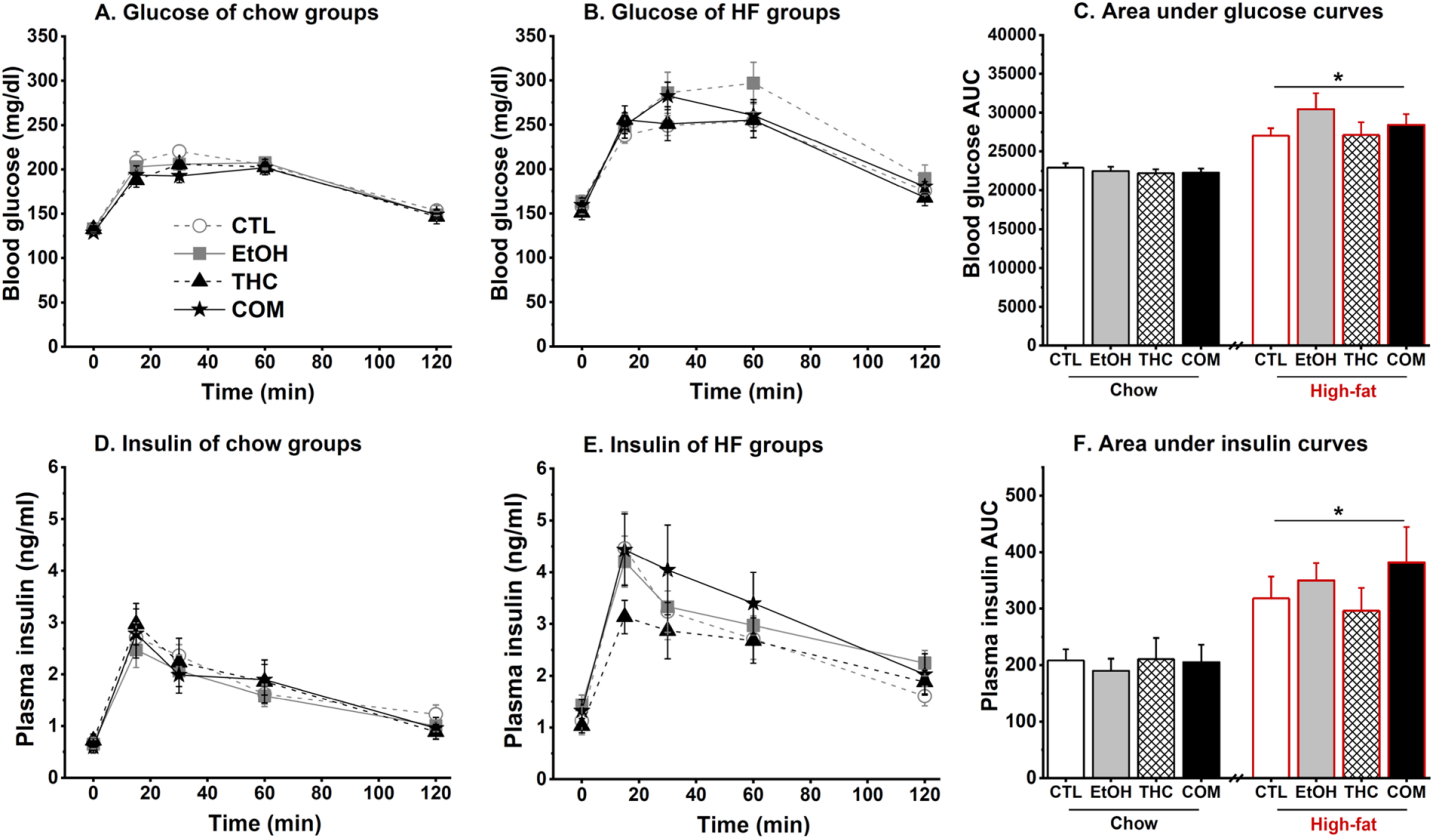
Effects of oral THC, moderate alcohol, and diet on glucose tolerance (Chow, n = 10–12/group and HFD, n = 7–8/group). Prior drug exposure had no effect on glucose clearance in rats (**A**) perpetually maintained on chow diet, and those (**B**) that consumed HFD for 4 days (P67–P70) after the diet switch (see Fig. 1B). (**C**) Area under curve revealed that 4-day HFD consumption altered glucose tolerance with no significant effect of prior drug exposure: *p < 0.0001. Prior drug exposure had no effect on insulin release in response to the oral glucose loads in both groups of rats that were (**D**) perpetually maintained on chow diet, and those (**E**) that consumed HFD for 4 days (P67–P70) after the diet switch. (**F**) Area under curve revealed that 4-day HFD consumption potentiated insulin release with no significant effect of prior drug exposure: *p < 0.0001.

**Figure 6:**
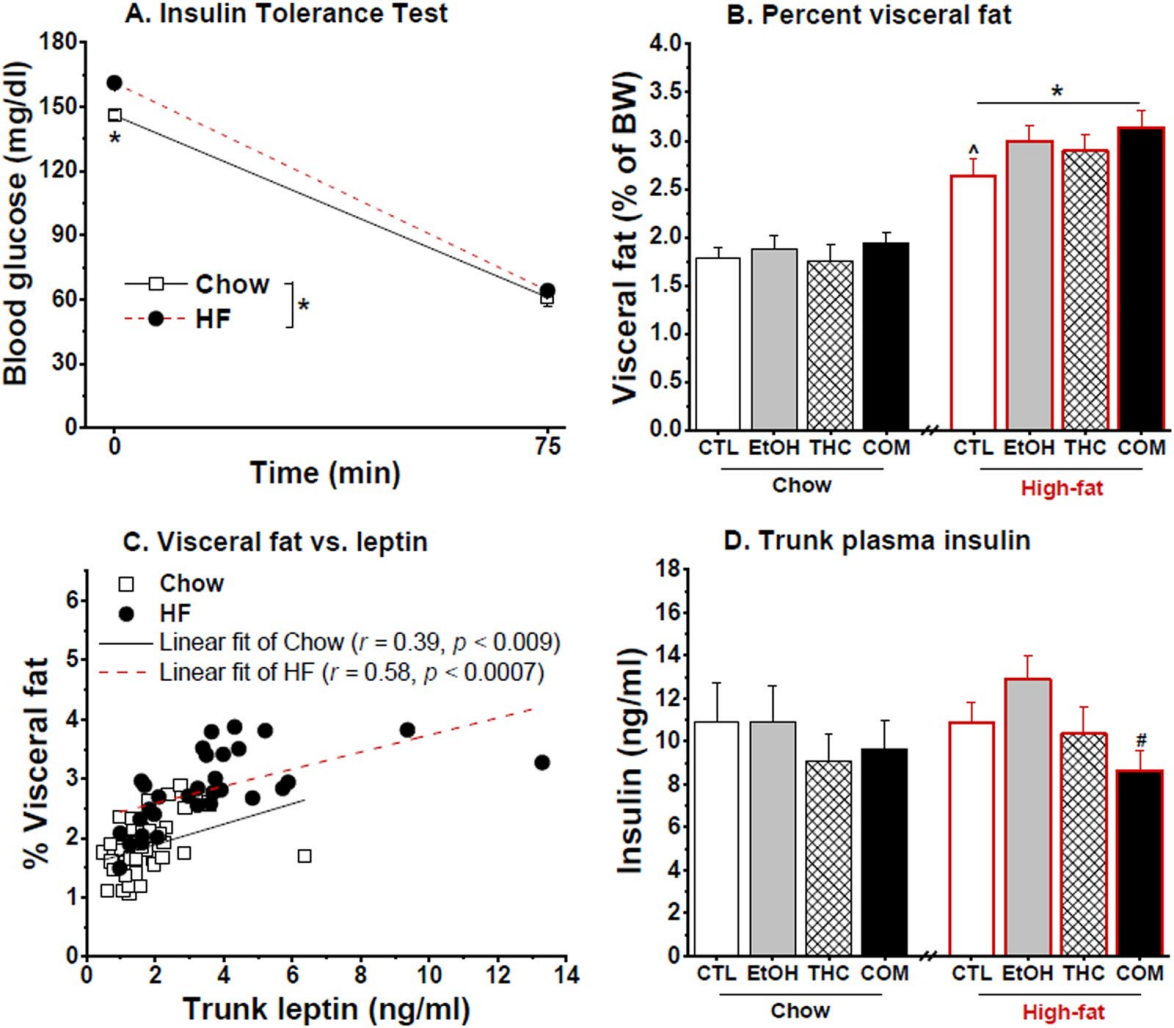
Effects of oral THC, moderate alcohol, and diet on some metabolic parameters (Chow, n = 10–12/group and HFD, n = 7–12/group). Nine days of HF diet feeding (**A**) increased fasting blood glucose with no effect on insulin sensitivity during the insulin tolerance test: *p < 0.002, (**B**) increased visceral fat composition: *p < 0.0001, (**C**) increased plasma leptin concentration: *p < 0.004, and (**D**) had no effect on plasma insulin concentration. Visceral fat composition positively correlated with leptin measured in trunk plasma in both the chow- (*r* = 0.39, *p* < 0.009) and HFD-fed (*r* = 0.58, *p* < 0.0007) groups. Prior alcohol or oral THC consumption did not significantly influence the above measures. However, under the HFD condition, *t*-test revealed that the COM rats tended to have higher visceral adiposity compared with the CTLs: ^*p* < 0.06 and lower trunk insulin compared with the EtOH group ^#^*p* < 0.007.

The HFD consumers had elevated basal blood glucose (Fig. 5A,B) and insulin concentrations (Fig. 5D,E) relative to the chow consumers following a mild fast [*F*_(1,24)_ = 42.25 and 28.92, respectively; both *p* < 0.0001]. During the OGTT, three-way repeated-measures ANOVA uncovered no effect of drug treatment and diet on blood glucose clearance and plasma insulin concentrations across time. The HFD groups demonstrated greater area under curve (AUC) of blood glucose [*F*_(1,24)_ = 78.52, *p* < 0.0001; Fig. 5C] and plasma insulin [*F*_(1,24)_ = 32.78, *p* < 0.0001; Fig. 5F] concentrations compared with the chow groups. Within each diet condition, there were no effects of prior alcohol and THC exposure on the metabolic parameters. Because there was no effect of prior drug exposure on blood glucose levels during the ITT, we collapsed the groups by diet condition. The HFD-fed groups had higher fasting blood glucose concentrations compared with the chow-fed groups [diet × time effect: *F*_(1,88)_ = 4.61, *p* < 0.04; post hoc, *p* < 0.002; Fig. 6A]. There was, however, no diet effect on blood glucose level at the 75-min time point – indicating that the nine-day HFD consumption did not alter insulin sensitivity in the rats. Furthermore, the HFD-fed rats had higher percent visceral fat [*F*_(1,38)_ = 84.56, *p* < 0.0001; Fig. 6B] and trunk plasma leptin concentration [*F*_(1,23)_ = 10.48, *p* < 0.004; Fig. 6C], with no effect of prior drug use. Percent visceral fat was positively correlated with plasma leptin in both the chow- [*r* = 0.39, *p* < 0.009] and HFD-fed [*r* = 0.58, *p* < 0.0007] groups. Under the HFD condition, and mirroring the body weight results, the COM group had slightly higher percent visceral fat compared with the CTLs [*t*_(20)_ = −2.02, *p* < 0.06]. Neither drug treatment nor diet condition influenced trunk insulin levels taken 75 min after the insulin injection (Fig. 6D). Finally, a *t*-test based on our *a priori* hypothesis that co-use of alcohol and THC would produce distinct effect from the use of either drug alone revealed that, under the HFD condition, the COM group had significantly lower trunk insulin levels than did the EtOH group [*t*_(21)_ = 3.02, *p* < 0.007; Fig. 6D].

## Discussion

Our data reveal that 16 days of separate and combined alcohol and THC exposure can have distinct effects on subsequent glucose and insulin homeostasis in young male rats. Experiment 1 showed that compared to the CTL, EtOH, and THC groups, the COM rats had the lowest insulin levels to clear glucose during an OGTT that occurred during abstinence. The seemingly increased insulin sensitivity corroborated with low weight gain and the lowest visceral fat in the COM rats among all treatment groups. Experiment 2 investigated glucose and insulin homeostasis under different dietary exposures following longer abstinence from edible THC and alcohol co-use. Although the influences of adolescent drug use on glucose metabolism disappeared after approximately one-month of forced drug abstinence, the COM rats gained the most body weight and visceral fat after a 9-day HFD challenge. The findings of the two experiments suggest that the beneficial effects of moderate alcohol and THC co-administration on glucose and insulin homeostasis manifest soon after 16 days of drug exposure and no benefit is obtained as the length of abstinence increases. Importantly, they highlight that HFD consumption can reverse the beneficial effects of THC and moderate alcohol on energy metabolism and homeostasis.

Under the chow feeding condition, rats that received subcutaneous THC injections (THC or COM group) had reduced adiposity and plasma insulin concentrations (Experiment 1); and those that consumed THC via cookies showed no changes in those parameters (Experiment 2). In addition to the different time points of the adiposity assessment, the differing pharmacokinetic profiles of injected versus oral THC may also contribute to distinct results of the two experiments. The consumption of edibles prepared with marijuana extract is popular among medical and recreational marijuana users^27^. Consumption of THC via cookie infusion used in Experiment 2 is more reflective of how the drug is consumed today. Past studies show that humans who consumed THC via an edible product can display more than one peak plasma THC concentration; and the duration of the effects of the THC can be longer when it is consumed orally versus when it is inhaled or delivered intravenously^28^. However, due to first-pass hepatic metabolism, plasma levels of THC are lower when THC or cannabis is consumed orally compared to when smoked or injected^24,28,29^. Overall, unlike smoked or parenterally administered THC, the bioavailability of orally consumed THC tends to be low and more erratic.

The effects of THC on glucose homeostasis in humans can depend not only on the administered dose, but also on the route of administration^19^. One study showed that intravenous THC impaired glucose tolerance but smoked THC had no effect. Such observations may be explained by the subjects’ prior experience with marijuana smoking^19^, by the fact that the bioavailability of THC is slightly greater following intravenous cannabis administration compared with inhalation^30^, or by unknown variables that await elucidation. Epidemiological reports on the metabolic effects of alcohol or marijuana often statistically control for the use of other drugs or screen for and exclude participants with a history of poly-substance use. Ours is the first preclinical rodent study to 1) investigate the effects of THC administered parenterally and via an edible product on subsequent glucose homeostasis and insulin sensitivity, and 2) examine the long-term effects of adolescent alcohol and oral THC co-exposure on feeding behavior and metabolic outcomes during young adulthood. We demonstrated that moderate daily use of alcohol or THC alone had no significant effects, but co-use of the two can produce additive or even opposite effects on circulating insulin levels and adiposity (Figs 2 and 6).

Alcohol and THC can interact in unique and complex ways to affect physiology. A past study demonstrated that smoking cannabis after drinking alcohol can reduce BECs attained and the psychoactive effects of alcohol in humans^31^. However, others have observed no effect of smoked THC on BEC^32^. Intriguingly, other human studies found that drinking before smoking or vaporizing cannabis increases blood levels of THC^33,34^. In this and our previous study^24^, subcutaneous or oral THC did not alter BEC in rats. We also observed that moderate alcohol consumption following subcutaneous THC injection resulted in lower plasma levels of the active THC metabolite 11-hydroxy-THC^24^. Our preliminary finding calls for further investigation of the long-term effects of alcohol and cannabis co-use on glucose homeostasis, including studies to decipher the cellular and pharmacokinetic mechanisms that mediate the outcomes.

In consonance with our previous findings^24^, subcutaneous and oral THC differently affect saccharin and alcohol intake. Whereas subcutaneous THC had no significant effects, rats that consumed THC-laced cookies subsequently consumed less saccharin and alcohol solution during the last 3 h of the dark cycle. We have previously shown that oral THC can reduce alcohol intake to the extent of complete avoidance^24^. By contrast, subcutaneous and oral THC had similar effects on food intake and body weight. Except for extremely high doses e.g., 20 mg/kg^24^, neither route of THC administration altered daily calorie intake. Repeated administration via either route modestly suppressed weight gain during treatment. These findings concur with those of other researchers^35,36^. There is notable individual variation in the effect of THC on appetite since not all marijuana users overconsume sweet or calorie-rich foods when *high*^37^. Epidemiological research has also uncovered that chronic marijuana users can either be of normal weight or have lower body mass index or waist circumference compared with never-users, prompting opinions about the possible weight-reducing effect of cannabis^18,38^. Furthermore, a very low dose (0.001 mg/kg) of ∆^8^-THC, which is supposedly a more stable and potent CB_1_R agonist than ∆^9^-THC, stimulated food intake but did not promote weight gain in mice^39^. Notwithstanding, our observed null effect of oral THC on daily caloric intake goes against the acclaimed hyperphagic effects of cannabis or THC^13,35,40^. Our findings, instead, reinforce the conclusion that depending on its interaction with CB_1_R on GABAergic or glutamatergic neurons, THC can produce opposite effects on appetite^41^. The complexities of cannabinoid signaling in the brain feeding circuits, sensory systems, and peripheral tissues involved in energy balance regulation are yet to be untangled, and research efforts in these domains have blossomed in recent years^41–43^.

Contrary to our hypothesis that the impact of alcohol and THC on the adolescent brain will facilitate future overconsumption of caloric dense foods, we observed that separate or combined alcohol and THC exposure did not exacerbate hyperphagia on a palatable 45% HFD during abstinence. This contrary observation can be due to several factors. First, although researchers who have used similar adolescent THC exposure timelines (parenteral administration) observed persistent neural and behavioral abnormalities during abstinence^36,44^, our 16-day oral drug exposure duration may have been insufficient to elicit sustained brain changes that can engender abnormal feeding behavior during abstinence. Second, any brain abnormalities caused by drug treatments might have been mitigated during the three-week abstinent/washout period. Studies have observed brain-region-specific downregulation and desensitization of CB_1_R following chronic alcohol or THC exposure that resolved to varying extents during abstinence^36,45,46^. Hence, a shorter washout period or even an earlier onset and simultaneous consumption of HFD with drug treatment may uncover robust drug/diet interaction. Third, the type of HFD (45%) used and the short duration (nine days) of high-fat diet exposure may have precluded observation of overeating and adverse long-term effects of adolescent drug exposure on energy metabolism^47,48^. Prolonged exposure to HFD alters the organization and function of neurons and glia in the hypothalamus to promote obesity and metabolic dysfunctions^49^. An exposure that extends for a longer period may exacerbate the higher weight gain and visceral fat observed in the COM rats to reveal dysregulation of glucose and insulin homeostasis.

An elevated fasting glucose concentration within normal physiological ranges is an independent risk factor for the later onset of type 2 diabetes in otherwise healthy male subjects^50^. In support of our *a priori* hypothesis in Experiment 2, pairwise comparison revealed that the COM rats that consumed HFD gained more weight and had higher visceral adiposities compared with the CTLs on the same diet. Although the HFD-consuming rats in our study were not obese *per se*, it is plausible that the increased visceral adiposity may be associated with alterations to endocannabinoid tone in peripheral tissues like pancreas and visceral fat. Compared with non-obese humans, obese individuals have changes to the peripheral endocannabinoid tone^51,52^. We found that short-term (4–9 days) HFD feeding resulted in modest signs of glucose intolerance and elevated fasting blood glucose concentration, visceral adiposity, and plasma leptin concentration. It is worth noting that we compared the behavioral and metabolic effects of 45% HFD to that of standard chow diet. Use of control low-fat diet (LFD) is a preferable option for metabolic studies^53^, but standard chow diet (SD) can be a bona fide substitute when low-fat diet is not available. This point is buttressed by the recent observation that an 18-week consumption of SD or LFD elicited similar phenotypic, behavioral, and metabolic effects in rodents^48^. More follow-up studies on the effects of moderate alcohol and THC use on energy balance and glucose homeostasis are warranted.

We also observed insignificant effects of drug or diet on insulin sensitivity assessed via the ITT. Besides the fact that we should have measured blood glucose level at least once before the 75-min time point post insulin injection during the ITT, the 1 U/kg insulin dose we administered to the rats may have been too high to reveal subtle drug- or diet-induced changes in insulin sensitivity. Alcohol exposure or cannabinoids acting via CB_1_/CB_2_ receptors can stimulate or augment insulin secretion from pancreatic β-cells^22,40,54,55^. Furthermore, prolonged HFD consumption induces insulin resistance and hyperinsulinemia^25^. High or low insulin concentration under basal conditions reflects an imbalance in the amount or rate of insulin secretion and degradation by insulin degrading enzymes in the liver and kidneys. Compared with chow-fed rats, we found that those exposed to HFD expressed modest hyperglycemia and hyperinsulinemia following a mild fast and during the OGTT. However, HFD feeding had negligible effects on plasma insulin levels when measured 75 min after exogenous administration of 1 U/kg insulin (Fig. 6A). The differing outcome of insulin levels during the OGTT and following the ITT suggest that 1 U/kg bolus of exogenous insulin may differently affect insulin secretion and/or degradation in chow and HFD rats. Considering that alcohol, cannabinoid, and HFD exposure can compromise hepatic enzyme function to promote hyperinsulinemia and impair insulin signaling^56,57^, our findings counter the expectation of increased trunk insulin level in the HFD-fed COM group. Instead, our *a priori* comparisons revealed that the HFD-fed COM group did have lower insulin concentrations compared with the EtOH group (Fig. 6D). Other researchers have posited that cannabinoids can have opposing effects on insulin secretion in *ex vivo* pancreatic islet preparations^58^, and the effect of alcohol on insulin secretion is unclear^55^.

In summary, we aimed to investigate how THC alone or when combined with moderate alcohol during adolescence will affect subsequent glucose and insulin tolerance during young adulthood in male rats. Our data show that combined subcutaneous THC and alcohol drinking can distinctly reduce the amount of insulin needed for the proper clearance of an oral glucose load compared with the effect of either drug alone. We also noticed that following approximately one-month of abstinence from edible THC and alcohol co-use, glucose and insulin tolerance were normalized to that of subjects without drug exposure. Metabolic challenge with an HFD revealed a drug-diet interaction such that rats that co-used THC and alcohol (COM) gained the most weight and visceral fat. These HFD-fed COM rats also appeared to have faster clearance of an exogenous insulin injection than the EtOH rats with the same HFD challenge. Thus, we provide preliminary evidence to show that THC and alcohol co-exposure can distinctly alter the physiology of glucose and insulin homeostasis in a preclinical rodent model. Research on the effects of alcohol and THC co-exposure in females, during other developmental stages, and with other rodent strains will show whether the effects we observed are universal. Such research will also provide a glimpse into how the drugs could impact metabolic health in human populations.

## Materials and Methods

### Subjects

The subjects comprised of a total of 139 male Long-Evan rats (Envigo, Indianapolis, IN, USA) received on postnatal day 22 (P22). They were semi-pair housed in large polyethylene tubs with transparent Plexiglas cage dividers that enabled measurement of food and fluid intake for each rat. The colony was maintained in a temperature and humidity-controlled vivarium on a 12-h light/dark cycle (lights on at 11:30 AM). During habituation and drug exposure, rats had *ad libitum* access to a standard rodent chow (3.1 kcal/g; 58% carbohydrate, 24% protein, and 18% fat from soybean oil; 2018 Teklad global rodent diets 2018, Indianapolis, IN, USA). Tap water was provided in glass bottles fitted with stainless steel sippers. Daily animal handling and care occurred at 8:00 AM when body weight, food, and water intakes were recorded. All study protocols were approved by the Institutional Animal Care and Use Committee (IACUC) at the University of Illinois at Urbana-Champaign, and they conformed to the guidelines stipulated in the *Guide for the care and use of laboratory animals* by the National Research Council, 2011.

### Test fluids (saccharin, alcohol) and ∆^9^-tetrahydrocannabinol (THC)

Saccharin (0.1%) solution and sweetened 10% or 5% ethanol solution (v/v) were prepared as we previously described^24^. The appropriate volume of 200 proof alcohol was mixed in 0.1% saccharine solution to make the sweetened alcohol solutions. Test fluids were presented at home cages in plastic bottles fitted with stainless steel sipper tubes that minimize spillage and evaporation. THC for subcutaneous injection or cookie infusion (Goldfish Grahams Fudge Brownie, Pepperidge Farm; Norwalk, CT, USA) was prepared by suspending the contents of 10 mg dronabinol capsules (Actavis Pharm, Inc.; Parsippany, NJ, USA) in a sesame oil vehicle^24^.

### Experiment 1: Procedures

Forty male rats were the subjects of Experiment 1. The experimental procedures that we employed are detailed in Experiment 1a of our past publication^24^. Briefly, following habituation and drinking training, the rats were divided into four groups (n = 10/group) by matching average body weight and saccharin intake: control (CTL) given saccharin and oil injection, ethanol (EtOH) given 10% ethanol (v/v) and oil injection, THC given saccharin and THC injection, and combination (COM) given 10% ethanol and THC injection. At 8:30 AM from P30–P45, animals received daily subcutaneous THC or oil injections immediately before test fluid access in their home cages. Chow and test fluid intakes were measured at 11:30 AM (3-h intakes) when test fluids were supplanted by tap water. Drug and test fluid exposures spanned 16 days (P30–P45). The 3 mg/kg/day dose lasted for eight days, while 5 and 10 mg/kg/day doses each lasted for four days^36^. One rat in the EtOH group was discovered dead at 8:00 AM on P42. Another EtOH rat was accidentally injected with 10 mg/kg THC on P43. The subsequent data from this rat were excluded from analysis. Chow and water were freely available for one week after the last drug exposure day. The experimental timeline is summarized in Fig. 1A.

### Oral glucose tolerance test (OGTT) and sacrifice

Following an overnight 16-h fast, we performed the OGTT on P54 (Fig. 1A) according to our established protocol^23^. After fasting blood glucose was measured using an AlphaTRAK glucometer (Abbott Labs) and blood was collected using heparinized microcapillary tubes via a tail clip, a load of 2.0 g/kg 20% glucose solution was administered through oral gavage. Blood glucose levels were measured, and tail blood was collected at 15, 30, 60, and 120 min post glucose gavage. The blood samples were centrifuged at 1,000 × g for 15 min at 4 °C, and the plasma was collected and stored at −80 °C for later insulin measurement using an ELISA kit (ALPCO, Salem, NH, USA) as we have previously done^23^. Within 1 h after the OGTT, the animals were sacrificed, and their carcasses stored at 4 °C overnight. The epididymal and retroperitoneal fat pads of each rat were dissected and weighed by researchers blinded to the group assignment. Visceral fat composition was calculated with reference to the body weight on the day of sacrifice^59^.

### Experiment 2: Procedures

Drug training and exposure procedures were performed, as we have previously described^24^. The 99 rats were first habituated to the vivarium for four days. From P26–P29, the animals were trained to consume 0.1% saccharin along with chow during the last 3 h of the dark cycle (8:30–11:30 AM). Following measurement of food and fluid intakes, the first and second oil-laden cookies were respectively provided at 12:30 PM and 9:00 PM. At the end of training, rats were assigned to one of four groups: control (CTL, n = 25) given saccharin and oil-laden cookies, ethanol (EtOH, n = 24) given 5% ethanol (v/v) and oil-laden cookies, THC (n = 24) given saccharin and THC-laden cookies, and combination (COM, n = 26) given 5% ethanol and THC-laden cookies. During the 16-day treatment period, rats first had 3-h access to saccharin or 5% alcohol (v/v) solution followed by a cookie laced with oil or THC (1.5, 3, or 5 mg/kg) presented twice per day at 12:30 PM and 9:00 PM (Fig. 1B). Each cumulative daily dose of 3, 6, 8, and 10 mg/kg THC lasted for four days. To ascertain how oral THC would affect blood ethanol concentration (BEC) in these rats, tail blood was sampled at 1-h during a day of 8 mg/kg/day THC consumption, and at 3-h during a day of 10 mg/kg/day THC consumption. BEC was analyzed in those samples using an ELISA kit (Cell Biolabs, San Diego, CA, USA) as we previously described^24^.

### High-fat diet exposure and oral glucose tolerance test (OGTT)

Following three weeks abstinence, rats in all four groups were subdivided into a chow group that continued consuming regular chow diet (CTL and COM, n = 14/group; EtOH and THC, n = 12/group) or a high-fat diet (HFD) group whose diet was switched to a 45% HFD (4.73 kcal/g; 45% fat from lard and soybean oil, 35% carbohydrate, and 20% protein; D12451, Research Diets, New Brunswick, NJ, USA; CTL, n = 11; EtOH, THC, and COM, n = 12/group). On the fourth day of this diet regimen (P70), rats were overnight food-restricted such that their intake was 70% of their respective intake on P69. OGTT was performed in the middle of the light cycle (between 2:00 and 7:00 PM) on P71 according to our established protocol^23^ and as described in Experiment 1 above.

### Insulin tolerance test (ITT) and tissue harvest

After the OGTT, rats were returned to their home cages to resume *ad libitum* access to their respective diets for four more days (P71–P74). On P75, they were mildly food-restricted to 90% of their average free-feeding intakes on previous two consecutive days (P72 and P73) following the OGTT. Between 2:00 and 7:00 PM on P76, fasting blood glucose levels were measured before rats were intraperitoneally injected with 1.0 U/kg of insulin (Humulin R, U-100; Lilly USA, LLC, Indianapolis, IN, USA). The 1.0 U/kg insulin dose was chosen based on its use by other researchers to assess systemic insulin sensitivity^60^. Seventy-five min later, blood glucose was measured before rats were rapidly decapitated. Trunk blood was collected into EDTA-coated tubes, centrifuged and plasma was collected to measure plasma levels of insulin (ALPCO, Salem, NH, USA) and leptin (Cat. *#*90040; Crystal Chem, Elk Grove Village, IL, USA). Animal carcasses were stored at 4 °C overnight. Epididymal and retroperitoneal fat pads were dissected and weighed by researchers blinded to the group assignment. Percent visceral fat composition was calculated with reference to the body weight on the day of sacrifice^59^.

### Statistical analysis

Data are presented as mean ± standard error of the mean (SEM) and were analyzed by factorial ANOVA or repeated-measures ANOVA (Statistica 13.3; TIBCO Software Inc.; Palo Alto, CA, USA). Statistically significant main effects and interactions were accompanied with Tukey’s HSD post hoc tests. Comparison between two groups within a diet condition was conducted with independent samples *t*-test. The linear relationship between ingested alcohol dose and BEC, as well as between plasma leptin and percent visceral fat were analyzed by Pearson’s correlation. *P* < 0.05 was considered significant.

## Data Availability

The raw data of the results presented in this manuscript are available upon request.
